# Precipitation and carbon-water coupling jointly control the interannual variability of global land gross primary production

**DOI:** 10.1038/srep39748

**Published:** 2016-12-23

**Authors:** Yao Zhang, Xiangming Xiao, Luis Guanter, Sha Zhou, Philippe Ciais, Joanna Joiner, Stephen Sitch, Xiaocui Wu, Julia Nabel, Jinwei Dong, Etsushi Kato, Atul K. Jain, Andy Wiltshire, Benjamin D. Stocker

**Affiliations:** 1 Department of Microbiology and Plant Biology, Center for Spatial Analysis, University of Oklahoma, Norman, OK 73019, USA; 2 Institute of Biodiversity Science, Fudan University, Shanghai, 200433, China; 3 Helmholtz Centre Potsdam, German Research Center for Geosciences (GFZ), Telegrafenberg A17, 14473 Potsdam, Germany; 4 State Key Laboratory of Hydroscience and Engineering, Department of Hydraulic Engineering, Tsinghua University, Beijing, China; 5 Laboratoire des Sciences du Climat et de l’Environnement, CEA CNRS UVSQ, Gif-sur-Yvette 91190, France; 6 NASA Goddard Space Flight Center, Greenbelt, MD, USA; 7 College of Life and Environmental Sciences, University of Exeter, Exeter EX4 4RJ, UK; 8 Max Planck Institute for Meteorology, Bundesstr. 53, 20146 Hamburg, Germany; 9 Global Environment Program, Institute of Applied Energy (IAE), Minato, Tokyo 105-0003, Japan; 10 Department of Atmospheric Sciences, University of Illinois, Urbana, IL 61821, USA; 11 Met Office Hadley Centre, FitzRoy Road, Exeter EX1 3PB, UK; 12 Department of Life Sciences, Imperial College London, Silwood Park, Ascot SL5 7PY, UK

## Abstract

Carbon uptake by terrestrial ecosystems is increasing along with the rising of atmospheric CO_2_ concentration. Embedded in this trend, recent studies suggested that the interannual variability (IAV) of global carbon fluxes may be dominated by semi-arid ecosystems, but the underlying mechanisms of this high variability in these specific regions are not well known. Here we derive an ensemble of gross primary production (GPP) estimates using the average of three data-driven models and eleven process-based models. These models are weighted by their spatial representativeness of the satellite-based solar-induced chlorophyll fluorescence (SIF). We then use this weighted GPP ensemble to investigate the GPP variability for different aridity regimes. We show that semi-arid regions contribute to 57% of the detrended IAV of global GPP. Moreover, in regions with higher GPP variability, GPP fluctuations are mostly controlled by precipitation and strongly coupled with evapotranspiration (ET). This higher GPP IAV in semi-arid regions is co-limited by supply (precipitation)-induced ET variability and GPP-ET coupling strength. Our results demonstrate the importance of semi-arid regions to the global terrestrial carbon cycle and posit that there will be larger GPP and ET variations in the future with changes in precipitation patterns and dryland expansion.

Carbon uptake through photosynthesis by terrestrial ecosystems is the largest flux in the global carbon cycle^1^. This flux, also known as gross primary production (GPP), drives not only ecosystem functioning, but also terrestrial carbon sequestration, which currently offsets more than one third of anthropogenic CO_2_ emissions^2^. Over the past 50 years, an enhanced seasonal exchange of CO_2_ has been observed in the Northern Hemisphere^3^, which was interpreted as increasing GPP in northern ecosystems induced by CO_2_ fertilization, extended growing seasons, and nitrogen deposition^3^,^4^,^5^. However, the GPP fluctuations superimposed on this increasing trend are less studied. With the increasing frequency of extreme climate events^6^, the interannual variability (IAV) of GPP is also projected to increase^7^, and will cause significant impacts on the global terrestrial carbon cycle^8^. The IAV of carbon uptake was shown to have distinctive spatial patterns, with hotspots on semi-arid or grassland ecosystems^9^,^10^,^11^. Although these spatial patterns are often explained by the interannual variation of water availability in semi-arid ecosystems, specifically, the precipitation variability, biotic meristem growth potential and their interactions^11^, the underlying mechanism is not well established. A key limitation to understanding this phenomenon and its potential feedbacks is that no accurate or direct GPP measurements are available at the global scale: diagnostic models and prognostic models show large differences in the spatio-temporal mean and variability patterns of GPP^12^. Previous studies often use median or average values as model ensembles to reduce the uncertainties^13^. However, un-weighted ensembles are not suitable to characterize spatial patterns for each individual year for two reasons: median values only keep limited information and unweighted averages may lead to biases from outliers.

In recent years, significant progress was made to develop satellite-retrieved solar-induced chlorophyll fluorescence (SIF) datasets^14^,^15^ that offer a new proxy for direct quantification of plant photosynthesis at a global scale. SIF is a very small amount of energy emitted by vegetation during photosynthesis, and it is sensitive to the electron transport rate and the absorbed radiation by chrolophyll^16^. Empirical studies have shown that SIF is highly correlated with GPP in space and time^15^,^17^,^18^,^19^,^20^. Recent modelling studies also exhibited high correlation between SIF and GPP at daily or 8-day scales^21^,^22^. Therefore, SIF can be used as a benchmark to evaluate GPP model performance^23^.

In this study, our first objective is to improve the spatial patterns of global estimates of GPP. We developed a method to calculate a weighted ensemble of GPP from multiple models that best approximate spatial and temporal variations in SIF derived from GOME-2 instrument^14^,^24^. To improve global estimates of GPP as compared with a straight average of an ensemble of models, we provided a weighted ensemble estimate using three data-driven models (VPM^18^, MTE^25^, and MOD17^26^) and eleven process-oriented models from the TRENDY-v4 project^27^ (CLM4.5, ISAM, JSBACH, JULES, LPJ, LPJ-GUESS, LPX, OCN, ORCHIDEE, VEGAS, and VISIT, see Materials and Methods). The weight of each model was determined by how well its GPP matches the spatial patterns of its proxy, SIF, within each biome type, i.e., higher weights were given to more realistic models and vice versa (see Methods). Our second objective is to better understand what factors control the spatial patterns of GPP IAV using the resultant SIF-constrained grid-based GPP dataset. The IAV of GPP and Evapotranspiration (ET) were analyzed together since they are closely coupled. We also adopted the Budyko framework^28^ to explain the IAV pattern along the aridity index (hotspot in semi-arid regions).

## Results and Discussion

**Figure 1:**
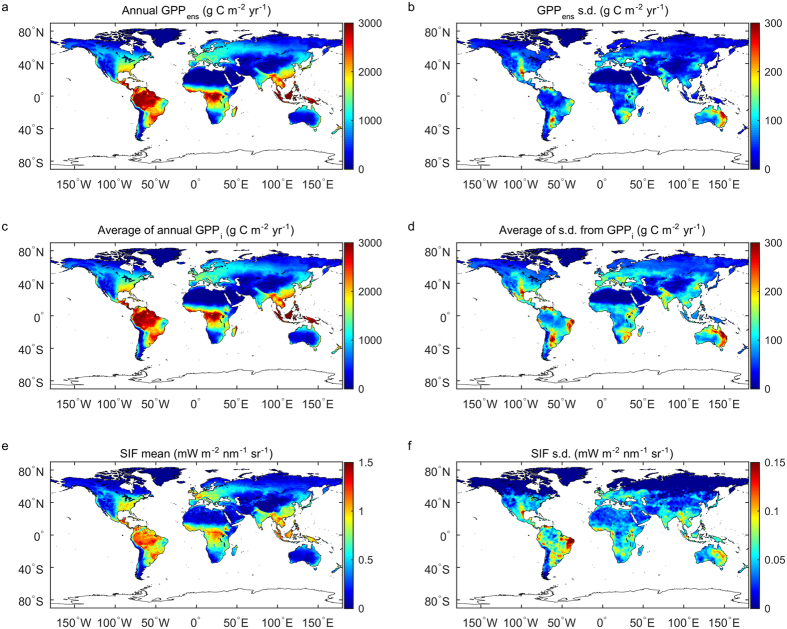
Mean and variability of GPP and SIF (2000–2011). (**a,b**) Mean and standard deviation (s.d., as a representative of variability) of annual GPP from ensemble of all models with the 2^nd^ order weight (GPP_ens_) for 2000–2011. (**c,d**) unweighted average of annual GPP and GPP s.d. calculated from each individual model (GPP_i_) for 2000–2011. (**e,f**) Annual mean SIF and SIF s.d. from GOME-2 for 2007 to 2015. The SIF variances were smoothed with a 3 × 3 pixel smoothing window after removing the error (see Supporting Information, Text S7). Maps were created using Matlab R2016a (http://www.mathworks.com/products/matlab/)

The annual mean and IAV of the weighted ensemble of GPP (GPP_ens_) show very good spatial consistency with the average of means and IAVs from individual models (GPP_i_) and GOME-2 SIF retrievals^24^ (Fig. 1). The weighted global mean GPP is 123.8 ± 1.6 Pg C yr^−1^ for data-driven models, 133.6 ± 1.9 Pg C yr^−1^ for process-based models, and 130.4 ± 1.7 Pg C yr^−1^ for all models (with ± being used for 1-sigma standard deviation). The weighted ensemble mean from data-driven models is very close to the previous estimates from multiple diagnostic models^1^ (~123 Pg C yr^−1^), the seasonal cycle of oxygen isotope measurements of atmospheric CO_2_^29^ (~120 Pg C yr^−1^), and a recent synthesis study^12^. The ensemble mean for all 14 models is slightly higher due to the relatively higher GPP estimates from the process-based models, possibly because these process models do not all represent nutrient limitation. The spatial patterns of mean annual GPP and IAV of GPP are similar for different weight orders and model groups (Supporting Information, Figs S7–S9).

**Figure 2:**
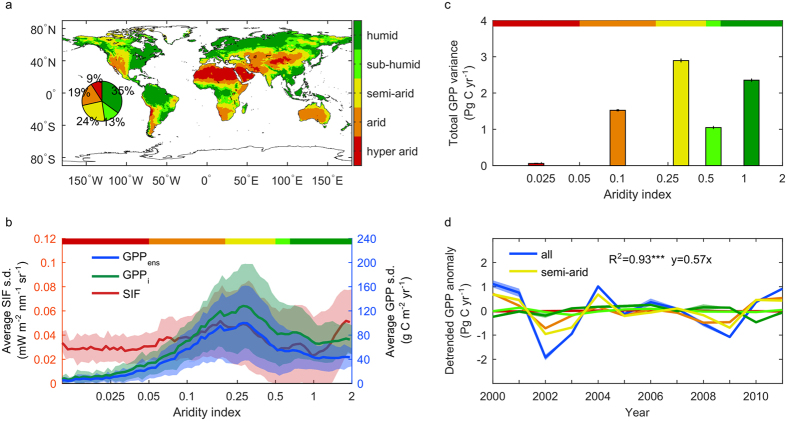
(**a**) The aridity map calculated as a ratio between precipitation from GPCC^55^ and potential evapotranspiration from MOD16 product^56^. Long-term averages from 2000 to 2013 for both products were used; the classification of aridity regions is based on UNEP (Supporting Information, Table S1). The small pie chart indicates the proportion of each class in the global land area. (**b**) IAV of GPP ensemble (GPP_ens_), average of GPP IAV from individual models (GPP_i_) and SIF IAV at different aridity indices, with the shaded area indicating one s.d. of variation. (**c**) The contribution of total GPP variability from different aridity classes. Error bars stand for one s.d. using different orders of ensemble. (**d**) Detrended GPP anomaly for global and 5 individual aridity regions, from 2000 to 2011. GPP ensemble comes from all models, and the shaded area indicates the range of s.d. from different orders of weight (1^st^, 2^nd^, 4^th^, see Methods). Maps were created using Matlab R2016a (http://www.mathworks.com/products/matlab/).

With this weighted ensemble of GPP from 14 models, we explore the linkage between the spatial patterns of GPP IAV and aridity index (Supporting Information, Table S1), i.e., the ratio of long-term annual precipitation to potential evapotranspirative demand. Unlike previous studies defining semi-arid regions by vegetation types^10^, we use the climatological definition from the Food and Agriculture Organization (FAO)^30^, where semi-arid regions are areas with the aridity index ranges from 0.2–0.5. Comparison with the aridity index shows that most of the areas with high GPP_ens_ IAV are located in semi-arid regions (Fig. 2b). The average of GPP variability from individual models (GPP_i_) and SIF versus aridity index confirms this relationship, with the average GPP variability reaching its peak at aridity index values of around 0.3, corresponding to semi-arid regions. SIF IAV from GOME-2 also shows high variability in semi-arid regions, but another peak in humid regions, the latter being a possible artifact of higher SIF errors under the influence of the South Atlantic Anomaly (SAA) in South America^31^ (Supporting Information, Fig. S10). Although humid regions defined by aridity index occupy a larger proportion of the global land surface (35%) and contribute a larger fraction of global GPP (~57%), the total variability of GPP of humid regions is smaller than that in semi-arid regions (Fig. 2c). In addition, the southern hemisphere contributes nearly 40% of the total GPP variability with only 25.8% of the global land area.

When decomposing the variability of annual GPP during 2000–2011 into a long-term trend and detrended anomalies, semi-arid regions contribute 57% of the total detrended GPP variance (Fig. 2d). This is in line with the results of Alhstrom *et al*.^10^ who used the model tree ensemble (MTE) data-driven GPP and TRENDY v3 process-based models. Similar results are also found if we use the ensembles from either only data-driven models or only process-based models (Supporting Information, Fig. S11). Our results also show an increasing trend in the GPP ensembles with large geographic differences. The humid regions contribute the largest proportion to the increase (42.4%), followed by semi-arid regions (29.0%). The GPP in humid regions is less sensitive to the climate anomalies, but may benefit from nitrogen deposition in mid-latitudes that makes CO_2_ fertilization more effective^32^ and from longer growing seasons in high latitudes^33^, therefore yielding a higher contribution to the trend than to IAV.

**Figure 3:**
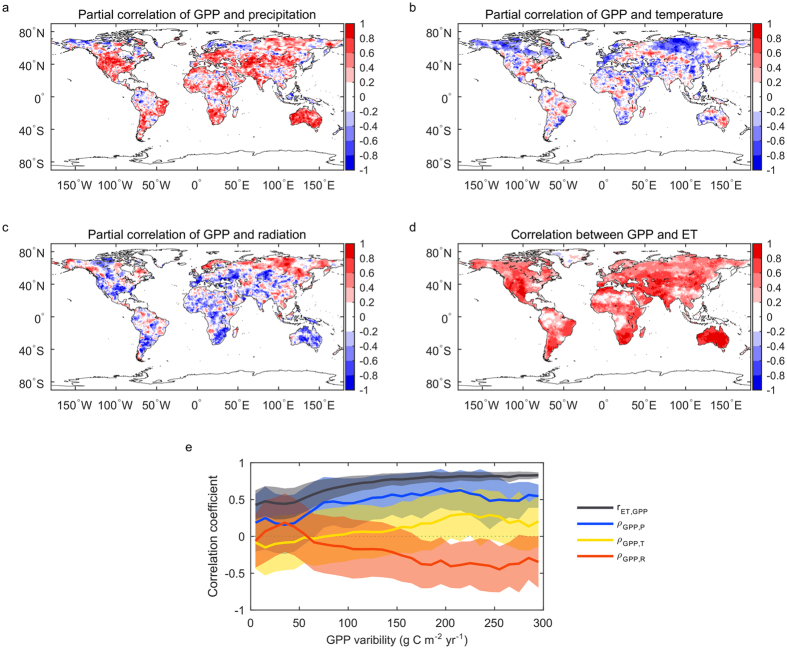
Relationship among GPP climate variables, and ET. Partial correlation (*ρ*) between annual GPP and (**a**) annual total GPCC precipitation, (**b**) annual mean CRU TS temperature and (**c**) annual sum CRUNCEP downward shortwave radiation with other two climate variable fixed. (**d**) averaged Pearson correlation (r) between GPP and all ET products (see Supporting Information, Text S2). (**e**) The relationship between 4 types of correlation versus GPP variability, with a bin size of 10 g C m^−2^ yr^−1^. Shaded areas represent the s.d. range within each bin. GPP is from the 2-order ensemble of all models and GPP ET correlation is from (**d**). Maps were created using Matlab R2016a (http://www.mathworks.com/products/matlab/).

We further attribute the IAV of GPP to different climatic factors by calculating the partial correlation coefficients between GPP and precipitation, temperature, and shortwave downward radiation. Similar to the results from a previous analysis using the MTE approach^1^, we find that for most land surfaces, annual GPP is controlled by precipitation, except for some tropical and boreal regions. Contributions from temperature and radiation are less significant (Fig. 3a–c). In addition, for most drylands (aridity index <0.65), GPP and ET are closely coupled, as represented by a high Pearson correlation coefficient (significant at the 0.05 level for 65.4% of areas in Fig. 3d). This tight coupling between GPP and ET in dry regions is robust to the choice of a particular ET dataset, as it is present for 12 different ET datasets (Supporting Information, Text S2). When plotting the correlation coefficients between ET and GPP against the GPP variability, the strength of the GPP-ET relationship increases with GPP variability (Fig. 3e), indicating that the changes in GPP in these high variability regions is closely linked to changes in ET. Precipitation is still the dominant climate factor for the large GPP variability, as represented by its highest partial correlation coefficient among the three major climate factors (Fig. 3e). Partial correlation between GPP and radiation is mostly negative due to the negative correlation with cloud cover and precipitation.

As GPP and ET are highly coupled in high GPP variability (semi-arid) regions, and both fluxes are closely linked to the precipitation anomalies, we use this empirical relationship to diagnostically investigate the factors related to high GPP IAV in semi-arid regions. One of the key factors that contributes to the GPP variability is the coupling strength or regression slope between GPP and ET (β). The contribution of this factor to the IAV of GPP is given by:


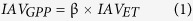


**Figure 4:**
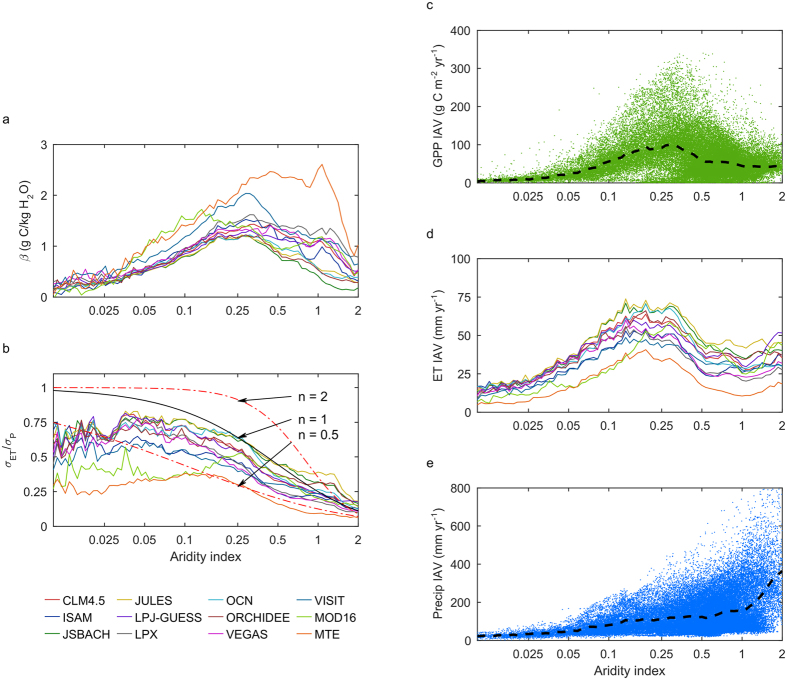
Decomposition of GPP IAV into ET IAV and precipitation IAV, and their relationship with aridity index. (**a**) Ratios of interannual anomalies of GPP to anomalies of ET (β) as a function of aridity index. Each line represents the mean value at each AI value. (**b**) Change in ET induced by change in precipitation, two dashed red line and the solid black line represent the prediction from Budyko framework with different n values. (**c**) GPP IAV from 2^nd^ order ensemble (γ = 2) of all models versus AI, the black dashed line represents mean value at each AI value. (**d**) ET IAV from all 12 ET products versus AI. (**e**) Precipitation IAV and its relationship with aridity index, the black dashed line represents the mean value at each AI value.

This equation only applies for regions where ET and GPP are strongly coupled on a temporal scale, i.e., high GPP variability areas as shown in the previous analysis (Fig. 3e). We calculate this β factor by using the linear regression slopes for each pixel between the weighted GPP anomalies and ET anomalies from 12 different ET products (Supporting Information, Fig. S12), and related it with the aridity index (Fig. 4a). These regression slopes generally increase with aridity index and can also be regarded as an approximation of the ecosystem water use efficiency (WUE) as long as the ecosystem is water limited^34^. The increasing trend of β with aridity index (i.e., from hyper arid to humid) is consistent with the fact that WUE increasing with precipitation across spatial gradients as suggested by a recent study using an inter-comparison of multiple models^35^. β starts to decrease when the aridity index approaches 1, where GPP and ET become decoupled from each other (Supporting Information, Fig. S13), i.e., when ecosystems are no longer water limited, decreases of GPP will not necessarily be linked to decreases of ET. When aridity index >1, some regions show relatively low β value; however, most of these regions are located in tropical forests where the ET-GPP correlation is low (Fig. 3d). Therefore, β is highest in arid to sub-humid regions and equation (1) is valid in these regions due to the close relationship between ET and GPP.

Given that the carbon and water fluxes are highly coupled in high GPP variability areas, we try to identify the climate regulation of ET variability and also GPP variability. Evapotranspiration is often thought to be physically constrained by the energy demand (potential evapotranspiration, PET) and water supply (precipitation, P) as described by the Budyko framework^28^,^36^ (see Methods). Based on this framework, the IAV of ET can be diagnosed from IAV of precipitation, given that the contribution of PET to IAV is negligible^37^, which is also confirmed by our analysis (Supporting Information, Fig. S14). Neglecting the contribution of PET is possible because precipitation variability in supply-(water) dominated regions is much larger than PET variability in demand-(energy) dominated regions, and this difference amplifies when we calculate the contributions from precipitation or PET exclusively using the derivatives of a Budyko function^38^ (Supporting Information, Fig. S15).

As ET variability is mostly dominated by P variability in high GPP IAV regions, we can investigate the precipitation contribution to the variability of ET. We get the spatial pattern of this sensitivity factor ((*σ*_*ET*_)/(*σ*_*P*_)) by calculating the ratio of ET variation to the precipitation variation (Supporting Information, Fig. S16). This sensitivity factor should, according to the Budyko framework (see Methods), decrease from 1 in hyper arid regions where all changes in precipitation leads to an equal amount change of ET, to 0 in extreme humid regions, where changes in precipitation do not affect ET (Fig. 4b). The relationship is also regulated by landscape and vegetation characteristics and by vegetation-precipitation feedbacks, as represented by the value of the exponent *n* in the Budyko equation (see Methods). Our data also show that (*σ*_*ET*_)/(*σ*_*P*_) decreases with increasing aridity index, and absolute (*σ*_*ET*_)/(*σ*_*P*_) values are relatively lower for the two data-driven ET products than for the process-based models. This is caused by the relative lower interannual variation of ET in data-driven products. Since the precipitation variability (*σ*_*P*_) increases with mean annual precipitation as well as with the aridity index, while the (*σ*_*ET*_)/(*σ*_*P*_) decreases with aridity index, ET variability (*σ*_*ET*_) reaches its highest values in arid to semi-arid regions (Fig. 4d). In addition, regression slopes between GPP and ET (β) are also highest in arid to sub-humid regions, both of which explain the highest GPP variability in semi-arid regions.

Many studies have shown the importance of precipitation to the global carbon cycle^1^,^39^. Previous studies hypothesize that the highest IAV of aboveground net primary production occurs in grasslands as a balance between the ecosystem potential productivity and the ecosystem stability^11^. Our study further documents a higher ET-GPP coupling (β) combined with a high precipitation-induced changes in water fluxes which is also predicted by Budyko framework in semi-arid regions. Using ET as an intermediate variable to connect the precipitation and stomata behavior which links both the carbon and water fluxes, we revealed how precipitation affects the IAV of GPP and ET. Because the highest ET variation in semi-arid regions is not only physically constrained by the precipitation and evapotranspiration ratio (fraction of precipitation that goes to evaporation, usually in the range from 0 to 1), but also affected by the vegetation coverage^40^. It is likely that the recent greening trend of vegetation, especially in northern hemisphere^41^,^42^, will also affect the peak of ET through the change of *n* in the Budyko equations. In addition, the elevated CO_2_ concentration in the atmosphere will affect the stomata behavior and water use efficiency^43^, which may further affect the peak of GPP variability in the space of aridity index. These issues still need further investigation by future studies.

GPP and ET variability are projected to increase in the future with changes in precipitation regimes and increases in evapotranspirative demand: the aridity map has changed over the past 60 years^44^, and drylands are projected to further expand in the future^45^. In addition, as the precipitation variability increases with global climate change, larger GPP and ET variability is expected in the future. On the other hand, with increased CO_2_ concentrations in the atmosphere, water will be more efficiently used and this tends to dampen the GPP ET relationship^43^. Grazing and frequent fire occurrence in semi-arid regions may also alter the value of GPP ET ratios, which makes this issue more complicated. Whether GPP IAV will continue to increase in the future still needs to be further assessed by state-of-the-art Earth system models and manipulated experiments.

**Figure 5:**
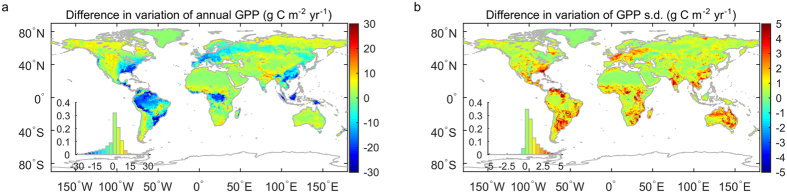
Difference in variation between the unweighted average and the weighted ensemble methods in terms of (**a**) annual GPP and (**b**) GPP s.d. Ensemble method with the order of two was used (γ = 2). The insets in the lower left corner show the histogram of the frequency distribution. All the units are in g C m^−2^ yr^−1^. Maps were created using Matlab R2016a (http://www.mathworks.com/products/matlab/).

In this study, we presented a weighted ensemble method to provide a better estimate of the GPP IAV from multiple models. By giving better performing models a higher weight, we expect to see an improved prediction of the GPP spatial patterns. Due to the spatial inconsistency between model gridcell and *in situ* measurements footprints^46^, we cannot directly verify the effectiveness of this method. However, this method is supposed to be more stable and robust to model selections, i.e., the variability of the GPP_ens_ IAV generated from different models will be smaller than that using a traditional unweighted averaging method. Figure 5 shows the difference of variability between the unweighted average and the weighted ensemble method using 13 out of 14 models as inputs (see Supporting information, Text S9). For annual GPP, the weighted ensemble method shows a relatively larger difference in tropical rainforest, south eastern U.S. and China. This may be caused by the relative large GPP discrepancy between data-driven models and process-based models in these regions, and the relatively higher weight factors given to the data-driven models. In terms of GPP s.d., we can see that the weighted ensemble method has smaller variation for almost all regions. This indicates the weighted ensemble method will give a more stable prediction of GPP s.d., which is also the focus of this study.

Global carbon cycle studies often rely on models to predict the spatial pattern of carbon fluxes^4^,^47^,^48^. However, large uncertainties still exist in different model groups (process-based or data-driven), or even within the same group. The uncertainties becomes more evident when analyzing the interannual variability patterns^12^. Our study presents a new framework to incorporate model simulations with global satellite observations to reduce these uncertainties of model outputs. Although GPP variability patterns produced by either process-based ensemble and data-driven ensembles are similar, we note that not all models captured these IAV patterns accurately, even models with similar distributions of annual GPP. With this precipitation-ET-GPP relationship being explained and the new ensemble framework developed, it provides a means to benchmark ecosystem models for water and carbon fluxes and their coupling.

## Materials and Methods

### GPP from 14 models

GPP dataset from 14 models for the period 2000 to 2011 can be grouped into two categories, i.e., three data driven models (VPM, MTE, and MOD17) and 11 process based models from TRENDY-v4 project. The VPM model and MOD17 are both light use efficiency models while using different driving data and parameters^26^,^49^. MTE uses machine learning algorithms which combine the observation from flux tower, satellite imagery, and gridded climate datasets^25^,^50^. The TRENDY-v4 datasets are the latest release for the TRENDY project which compared the simulation results from dynamic global vegetation models (DGVMs)^51^. All these models are driven by the same climate forcing data and monthly GPP estimates are used. (see Supporting Information, Text S1).

### Solar-induced chlorophyll fluorescence from GOME-2

The solar-induced chlorophyll fluorescence (SIF) measurements are retrieved from GOME-2 onboard the MetOp-A satellite. The GOME-2 instrument captures the Earth radiation from ~240 to 790 nm at a nominal nadir footprint of 40 × 80 km^2^ (and 40 × 40 km^2^ since 15 July 2013) with a spectral resolution of ~0.5 nm. Compared with SIF measurements from other satellites (GOSAT, OCO-2, etc.), GOME-2 data has the longest observation history (2007–2015). We used the version 26 SIF from Joiner *et al*.^14^,^24^ available at http://avdc.gsfc.nasa.gov. This product is retrieved at wavelengths surrounding 740 nm, where the far-red peak of SIF emission is located, based on a principal component analysis approach to account for atmospheric absorption. The initial results are quality controlled and aggregated to produce a level 3 monthly mean product at 0.5° × 0.5° spatial resolution^24^.

### The ensemble of GPP using SIF

Sun-induced chlorophyll fluorescence (SIF) is a small amount of energy released during the photosynthesis process^52^. When sunlight is absorbed by vegetation, a large proportion of energy goes to photosynthesis (known as photochemical quenching, PQ), and most of the remaining energy is dissipated as heat (known as non-photochemical quenching, NPQ); a small amount of energy (~1–2%) is reemitted as chlorophyll fluorescence. In cases of environmental stress, both PQ and SIF decrease while NPQ increases. Therefore, SIF can serve as a good proxy for plant photosynthesis.

The SIF product from the Global Ozone Monitoring Experiment 2 (GOME-2) that we used in this study has a satellite overpass time near 9:30 am local time. Therefore, for most areas, GPP should be correlated with SIF for each biome type, and the ratio between GPP and SIF should converge on a monthly scale. Hence, we can use SIF as a proxy for GPP within each biome type both spatially and temporally. To get the ensemble of all GPP estimates, the weight of each GPP product should reflect how well the GPP product matches its respective SIF spatial or temporal pattern within each biome type. Considering that SIF has relatively low values (0–4.0 mW m^−2^ sr^−1^ nm^−1^) compared to its uncertainties (0.1–0.4 mW m^−2^ sr^−1^ nm^−1^) at monthly scales, SIF can be averaged temporally (from monthly to yearly) or spatially (within-biome for each month) to reduce those uncertainties and compare with GPP. The land cover data used to identify different biome types came from MCD12C1^53^. This dataset has a spatial resolution of 0.05 degree with a proportion of each biome type for each grid cell. We aggregated it to 0.5 degree spatial resolution and recalculated the proportion of area for each biome type.

For the GPP ensemble using SIF as a proxy for spatial (temporal) variations, both SIF and GPP were temporally (spatially) averaged to get an annual map (a value for each biome type for each month). For each model *i*, the correlation between SIF and GPP for all pixels within each biome type *j* (for all 12 months for biome type *j*) is calculated as 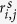 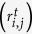.

The overall score of a model *i* (score_*i*_) is the weighted average of *r*_*i*, *j*_ by the area of each biome:


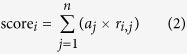


where *a*_*j*_ represents the biome type *j* as a fraction of the total area 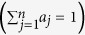. For each group (data-driven or process based or all-ensemble), the weight of *i*th model is calculated as:


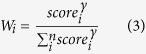


where *γ* is the order coefficient; in this study, we used 1^st^ order, 2^nd^ order and 4^th^ order to calculate *W*_*i*_. The ensemble GPP for each year is calculated as:


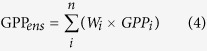


We chose the years 2007–2011 as the training period, due to the overlay of all process-based models, data-driven models, and availability of SIF from GOME-2. We applied the model contribution to the period 2000 to 2011 to get a GPP product at an annual scale for both spatial and temporal ensembles. Because we focused on the IAV of GPP, which is a spatial pattern, we only used the GPP ensemble with SIF as a spatial representative.

### The Budyko framework

The Budyko framework describes how energy availability (represented by potential evapotranspiration, PET or *E*_0_) and water availability (represented by precipitation, P) determine evapotranspiration (ET). It hypothesizes that the ratio of ET over P is a function of the ratio of PET over P, as follows:


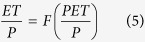


One analytical solution^38^ to this equation is:


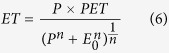


Where the parameter *n* is used to define different Budyko curves for landscape and vegetation characteristics. This analytical solution is similar to Fu’s solution^54^ and enables us to easily calculate the partial derivative of ET with respect to P. *n* ranges from 0.5 to 2, which covers most global landscape characteristics, and is used as a constraint to explain the ET and P IAV relationship. The IAV of ET (*σ*_*E*_) can be derived from this Budyko framework^37^:


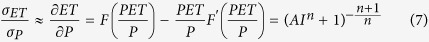


We use different *n* to get the possible distributions of the relationship between ∂*ET*/∂*P* and *P*/*PET* (aridity index).

## Additional Information

**How to cite this article**: Zhang, Y. *et al*. Precipitation and carbon-water coupling jointly control the interannual variability of global land gross primary production. *Sci. Rep.* **6**, 39748; doi: 10.1038/srep39748 (2016).

**Publisher's note:** Springer Nature remains neutral with regard to jurisdictional claims in published maps and institutional affiliations.

## Supplementary Material

Supplementary Information
