# Supplementary material for: Precipitation and carbon-water coupling jointly control the interannual variability of global land gross primary production
Source: Sci Rep. 2016 Dec 23;6:39748. doi: 10.1038/srep39748 (PMC5180184; doi:10.1038/srep39748)
Supplement: Supplementary Information [file srep39748-s1.pdf]

Supplementary Information for

Precipitation and carbon-water coupling jointly control the interannual variability of global land gross primary production

Yao Zhang[a,1], Xiangming Xiao[a,b,1], Luis Guanter[c], Sha Zhou[d], Philippe Ciais[e], Joanna Joiner[f], Stephen Sitch[g], Xiaocui Wu[a], Julia Nabel[h], Jinwei Dong[a], Etsushi Kato[i], Atul K. Jain[j], Andy Wiltshire[k], Benjamin D. Stocker[l]

[a]Department of Microbiology and Plant Biology, Center for Spatial Analysis, University of Oklahoma, Norman, OK 73019, USA

[b]Institute of Biodiversity Science, Fudan University, Shanghai, 200433, China

[c]Helmholtz Centre Potsdam, German Research Center for Geosciences (GFZ), Telegrafenberg A17, 14473 Potsdam, Germany

[d]State Key Laboratory of Hydroscience and Engineering, Department of Hydraulic Engineering, Tsinghua University, Beijing, China

[e]Laboratoire des Sciences du Climat et de l'Environnement, CEA CNRS UVSQ, Gif-sur-Yvette 91190, France

[f]NASA Goddard Space Flight Center, Greenbelt, MD, USA

[g]College of Life and Environmental Sciences, University of Exeter, Exeter EX4 4RJ, UK

[h]Max Planck Institute for Meteorology, Bundesstr. 53, 20146 Hamburg, Germany

[i]Global Environment Program, Institute of Applied Energy (IAE), Minato, Tokyo 105-0003, Japan

[j]Department of Atmospheric Sciences, University of Illinois, Urbana, IL 61821, USA

[k]Met Office Hadley Centre, FitzRoy Road, Exeter EX1 3PB, UK

[l]Department of Life Sciences, Imperial College London, Silwood Park, Ascot SL5 7PY, UK

Corresponding author: Yao Zhang (yaozhang@ou.edu),

Xiangming Xiao (xiangming.xiao@ou.edu)

S1. GPP products

- **VPM**

The Vegetation Photosynthesis Model (VPM) is a light use efficiency (LUE) model which has been tested over a variety of land cover types at CO_2 eddy flux tower sites (1-6). The GPP product we used in this paper is driven by the MODIS 8-day 500 m surface reflectance product (MOD09A1 C6), the MODIS 8-day 1 km Land surface temperature product (MOD11A2 C5), the MODIS land cover product (MCD12A2 C5), and the shortwave radiation and air temperature from the NCEP-DOE Reanalysis 2 dataset. The VPM (version 2.0) estimates GPP by using the product of absorbed photosynthetic active radiation by the chlorophyll ($APAR_{chl}$, calculated by $fPAR_{chl}$ × PAR) and the light use efficiency (LUE, ε_g):

$$\mathrm{GPP} = \varepsilon_g \times APAR_{chl} \tag{A1}$$

$$fPAR_{chl} = a \times (EVI - 0.1) \tag{A2}$$

Where the factor a = 1.25, and 0.1 in Eq. A2 is used to adjust EVI baseline (7, 8). The EVI is calculated from the MOD09A1 product and passed a rigorous quality check and gap-filling procedure (9). The LUE (ε_g) is reduced from a maximum light use efficiency (ε_0) by the temperature scalar (T_{scalar}) and a water scalar (W_{scalar}):

$$\varepsilon_g = \varepsilon_0 \times T_{scalar} \times W_{scalar} \tag{A3}$$

Detailed information about the model parameter estimation were documented in previous publications (1, 4, 10).

The GPP product at 8-day temporal resolution and 500 m spatial resolution was reprojected and aggregated into 0.5 degree (latitude and longitude). The annual GPP is calculated as the sum of each 8-day GPP over a Julian year, and the interannual variation of GPP is calculated below:

$$IAV = \sqrt{\frac{\sum_{i=1}^{n}(GPP_i - \overline{GPP})^2}{n}} \tag{A4}$$

Where GPP_i and $\overline{GPP}$ represent the annual GPP for year i and average annual GPP for year 2000 to 2011, respectively. n is the total number of years.

- **MPI-BGC (MTE)**

The MPI-BGC estimates GPP by upscaling global *in situ* CO_2 eddy flux observations with climate data and remote sensing fraction of absorbed photosynthetic active radiation (fAPAR) using a Model Tree Ensemble approach (11, 12). This dataset has a spatial resolution of 0.5° ×0.5°. This monthly dataset was first aggregated into annual sum and then the IAV over the period of 2000 to 2011 was calculated to match with other GPP products.

- **MOD17**

The MODIS GPP product (MOD17) employs a light use efficiency (LUE) approach to calculate GPP. The PSN model used in the MOD17 GPP product uses MODIS fPAR product as the fraction of PAR absorbed by vegetation for photosynthesis. MOD17 also uses vapor pressure deficit (VPD) as the water limitation of LUE and uses variable maximum LUE for individual biome types, which are determined by MODIS land cover map (MCD12). The MOD17 product (MOD17A2 C55) during 2000-2011 was downloaded from Numerical Terradynamic Simulation Group (http://www.ntsg.umt.edu/project/mod17#data-product) at the University of Montana. MOD17 GPP at 1-km spatial resolution and 8-day temporal resolution and was aggregated into 0.5 degree (latitude/longitude) to calculate annual GPP.

- **TRENDY-V4**

The TRENDY project compared simulation results from dynamic global vegetation models (DGVMs) under the same climate forcing data, with an objective to investigate the trend in net biome production (NBP) over the period 1980 to 2010 (13). The GPP simulation from 2000 to 2011 from the latest version TRENDY-V4 were used in our study. The model simulation is based on two experiments: S2, CO_2 and climate, S3, CO_2, climate and land use. In our study, the S2 simulation with variable CO_2 and climate forcing are used because not all models provide S3 simulations. Eleven models (CLM4.5 (14), ISAM (15), JSBACH (16), JULES (17), LPJ (18), LPJ-GUESS (19), LPX (20), OCN (21), ORCHIDEE (22), VEGAS (23), and VISIT (24)) from the TRNEDY project were used to calculate the inter-annual variability (IAV) of GPP for the period 2000 to 2011 (Fig. S1-S3). The spatial resolution of CLIM4.5 is 2.5° ×1.875°, JSBACH is ~1.875° ×1.875°, JULES is 1.875° ×1.25°, OCN and LPX are 1° ×1°. These annual GPP means and IAVs are calculated at the original resolution and spatially interpolated to 0.5° ×0.5° using cubic interpolation method.

S2. Evapotranspiration and potential evapotranspiration dataset

Evapotranspiration datasets from the model tree ensemble (MTE) (Jung et al. (12)), MODIS ET (27) (MOD16A3 C5), and 10 TREDNY models are used in our study (ET from LPJ is not used due to a data problem---abnormally low ET in tropical reigons). The MTE method integrates the eddy flux tower measured ET with remote sensing satellite images and meteorological data using a machine-learning algorithm (11). The MOD16A3 product calculates ET based on the Penman-Monteith algorithm (28) and was recently improved (27). We downloaded the potential evapotranspiration (PET) and evapotranspiration (ET) from Numerical Terradynamic Simulation Group website at the University of Montana (http://www.ntsg.umt.edu/project/mod16). The PET and ET datasets were annual sums at 1 km×1 km spatial resolution and we aggregated to 0.5° ×0.5°. ET from TRENDY-V4 were also interpolated into 0.5° ×0.5° spatial resolution. All ET datasets from 2000 to 2011 were used.

S3. Climate dataset

The GPCC V7 global precipitation at 0.5° ×0.5° is used in our analysis (29). This dataset has a monthly precipitation value from 1901 to 2013. The downward shortwave radiation was obtained from CRUNCEP V4 (http://dods.extra.cea.fr/data/p529viov/cruncep/) with a monthly sum and a spatial resolution of 0.5° ×0.5°. The monthly mean temperature was from CRU TS 3.23 (30) (https://crudata.uea.ac.uk/cru/data/hrg/cru_ts_3.23/) and also has a 0.5° × 0.5° spatial resolution. All the monthly climate datasets are aggregated to annual sums. The aridity index is calculated using GPCC precipitation over PET from MOD16 for the period 2000 to 2011. The classification scheme of the aridity regions based on aridity index is from UNEP (31) and can be found in Table S1. Because CRUNCEP V4 does not provide radiation after 2010, the climate correlation is calculated using data from 2000 to 2010.

S4. Aggregation of MODIS land cover map

In order to get the annual land cover map at 0.5° ×0.5° spatial resolution, we aggregate the MCD12C1 C5 product spatially. The aggregation is based on the average of area percentage of each biome type, therefore the output also includes percentage of each biome type for each grid cell. To make sure we can compare the SIF and GPP within each land cover type, we set a threshold to extract the grid cells with 'pure' land cover types. We set the threshold from 50% to 100%. With the increase of this threshold, the number of mixed pixels also increased and the available pixels decreased (Fig. S4). In this study, we used the 80% threshold, and only two biome types have less than 100 gridcells.

S5. Rationale of GPP ensemble using SIF as a reference

Recent studies have successfully retrieved SIF from satellites, and these SIF retrievals showed very good correlation with GPP from flux tower upscaled models and light use efficiency models (32). This close relationship between GPP and SIF is found to be higher within each biome type at monthly scale using data from both global simulation and *in situ* observations (33, 34). The Soil Canopy Observation of Photochemistry and Energy fluxes (SCOPE) model is a process-based model which can also simulate energy exchange within photosynthesis process, including SIF (35). Using this SCOPE model, recent studies investigated the relationship between GPP and SIF (36, 37). SIF_{yield} (defined as SIF/APAR, absorbed photosynthetically active radiation) shows a good linear correlation with photochemical yield (defined as GPP/APAR) when APAR is higher than 400 to 600 $\mu mol\ m^{-2}s^{-1}$. However, this linear relationship varies with V_{cmax}. If we assume the similar V_{cmax} within each biome type, SIF should have a good correlation with GPP at moderate or high light conditions relevant to satellite observations.

S6. Comparing GPP ensemble using SIF as a temporal proxy and spatial proxy

Because of the close relationship between SIF and GPP within each biome type, SIF can be used as a reference to evaluate the performance of various models in GPP estimation. To reduce uncertainties of SIF from GOME-2, we proposed two strategies to reduce uncertainties: monthly SIF is averaged either temporally to yearly average, or spatially within each biome types; and we then use temporally averaged SIF as spatial representative or the spatially averaged SIF as temporal representative of photosynthetic activity. The weights for each model from these two approaches are given in Figs. S5, S6. When using SIF as a temporal reference for each biome, the difference between each individual model is rather small (Table S2), and most models have relatively high correlation. This implies most models can simulate the seasonal variation of GPP relatively well. However, when using SIF as a spatial reference within each biome, the difference between each individual model is relatively large (Table S3), and many models have relatively low correlation coefficients. In addition, the interannual difference of the weight is very small (short error bar in Fig. S5), proving the method is relatively stable.

S7. Interannual variation of SIF

SIF data from the satellite have relative high noise because of the high spectral requirement for the sensor. For the GOME-2 dataset, the reported error for monthly mean gridded data are ~ 0.1-0.4 mW m^{-2} nm^{-1} sr^{-1} (26). This reported error is contributed from several sources: (1) the error for each individual SIF measurement, which is affected by the radiance noise, atmospheric conditions; (2) different observing conditions (view angle), sampling locations and time; (3) cloud contaminations; (4) various systematic errors. When calculating the interannual variation, the contribution from 2-4 is limited and is ignored in our analysis.

We use the following scheme to calculate the interannual variation of SIF:

1) For each gridcell each month, not only the aggregated SIF value (average of all valid SIF retrieval) is given, the GOME-2 SIF product also gives the number of individual SIF measurements used to aggregate the gridcell and the standard deviation of all retrievals. The uncertainty of this gridcell for month i (σ_i) will be the standard deviation of all retrievals (regarded as the uncertainty of each individual measurement) divided by $\sqrt{n}$, where n is the number of measurements used for aggregation. Therefore, we can get the uncertainty of SIF for each gridcell for each month.
2) From the SIF value and uncertainty for each month, we will further calculate the SIF mean and uncertainties for each year. Assuming for each gridcell, the uncertainty of SIF for month i (ε_i) follows a normal distribution ($\varepsilon_i \in \mathrm{N}(0, \sigma_i)$), where σ_i is the uncertainty which we calculated in the previous step, the uncertainty of annual average SIF for year j (ε_j) will also follow a normal distribution:

$$\varepsilon_j \in \mathrm{N}\left(0, \frac{\sqrt{\sum_{i=1}^{12} \sigma_i^2}}{12}\right) \quad (A5)$$

3) To calculate the actual IAV of SIF, we need to eliminate the uncertainty induced variability for each year ($\varepsilon_j \in \mathrm{N}(0, \sigma_j)$) from the calculated variability (σ_{cal}) for each gridcell. We found a very similar pattern of ε_j across different years. Because of the additivity of variance, the calculated IAV of SIF (σ_{cal}) can be explained by the real IAV of SIF (σ_{IAV}) and error of observation (σ_{ann}):

$$\sigma_{cal}^2 = \sigma_{IAV}^2 + \sigma_{ann}^2 \quad (A6)$$

where the error of observation (σ_{ann}) is calculated as follows:

$$\sigma_{ann}^2 = \sqrt{\frac{\sum_{j=2007}^{2015} \sigma_j^2}{9}} \quad (A7)$$

The spatial patterns of σ_{cal} and σ_{ann} are shown in Fig. S10.

S8. Decompose ET variation from precipitation variation and potential evapotranspiration variation

Using the Budyko framework, similar with equation (7) in the methods, we can also get the ET sensitivity to PET (E_0) change:

$$\frac{\partial ET}{\partial P} = \frac{1}{\left(1 + \left(\frac{E_0}{P}\right)^{-n}\right)^{\frac{n+1}{n}}} = (AI^n + 1)^{-\frac{n+1}{n}} \quad (A8)$$

$$\frac{\partial ET}{\partial E_0} = \frac{1}{\left(1 + \left(\frac{E_0}{P}\right)^{n}\right)^{\frac{n+1}{n}}} = (AI^{-n} + 1)^{-\frac{n+1}{n}} \quad (A9)$$

Therefore, ET variability can be decomposed into ET variability caused by precipitation variability (ET_P), and ET variability caused by PET variability (ET_{E_0}).

$$\mathrm{ET}_P = \frac{\partial ET}{\partial P} \times IAV_P \quad (A10)$$

$$\mathrm{ET}_{E_0} = \frac{\partial ET}{\partial E_0} \times IAV_{E_0} \quad (A11)$$

We calculate the ET_P and ET_{E_0} using different n values (0.5, 1, 2) to infer the ET change from change in precipitation and change in E_0. Results are shown in Fig. S15.

S9. Verification of the stability and robustness of the weighted ensemble method

To verify the stability and robustness of the weighted ensemble method to model inputs, we use 13 out of 14 models as input and test the variation of the annual GPP and GPP s.d. for both unweighted average method and the weighted ensemble method. Each time, we drop one model from the 14 models and use the rest 13 models as input. The GPP for each year is calculated as the average of the 13 model estimates for this year or using the ensemble method described in the method section. The annual GPP and GPP s.d. is then calculated from the output of each year by both methods. By repeating this process for 14 times, we can get all the possible combination of using 13 models as inputs. The variations of the annual GPP and GPP s.d. from 14 times run for both methods are regarded as the methods stability (Fig. S17).

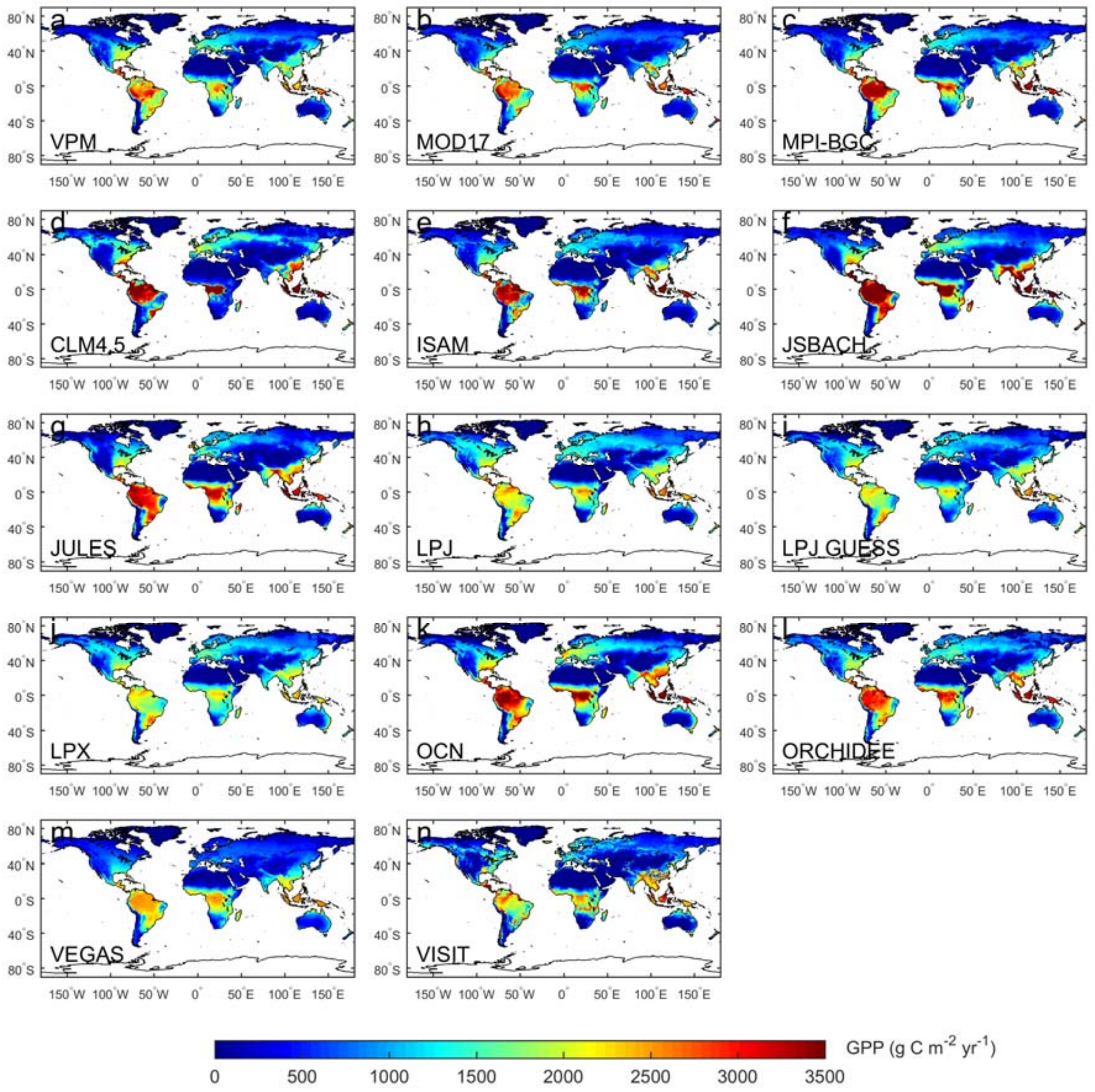

Fig. S1. Spatial patterns of annual mean GPP from 3 data-driven models, and 11 DGVMs from the Trendy v4 project. Maps were created using Matlab R2016a (http://www.mathworks.com/products/matlab/).

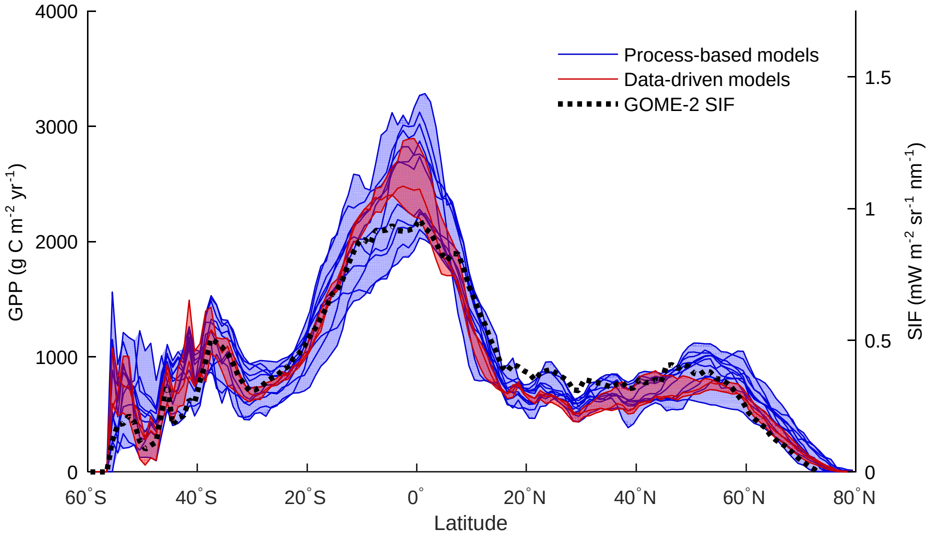

Fig. S2. Latitudinal pattern (1° bands) of annual GPP. The blue area represents the range of the DGVMs from the Trendy v4 project (CLM4.5, ISAM, JSBACH, JULES, LPJ, LPJ-GUESS, OCN, ORCHIDEE, TRIFFID, VEGAS, VISIT), the red area represents the range of the data-driven models (VPM, MTE, and MOD17).

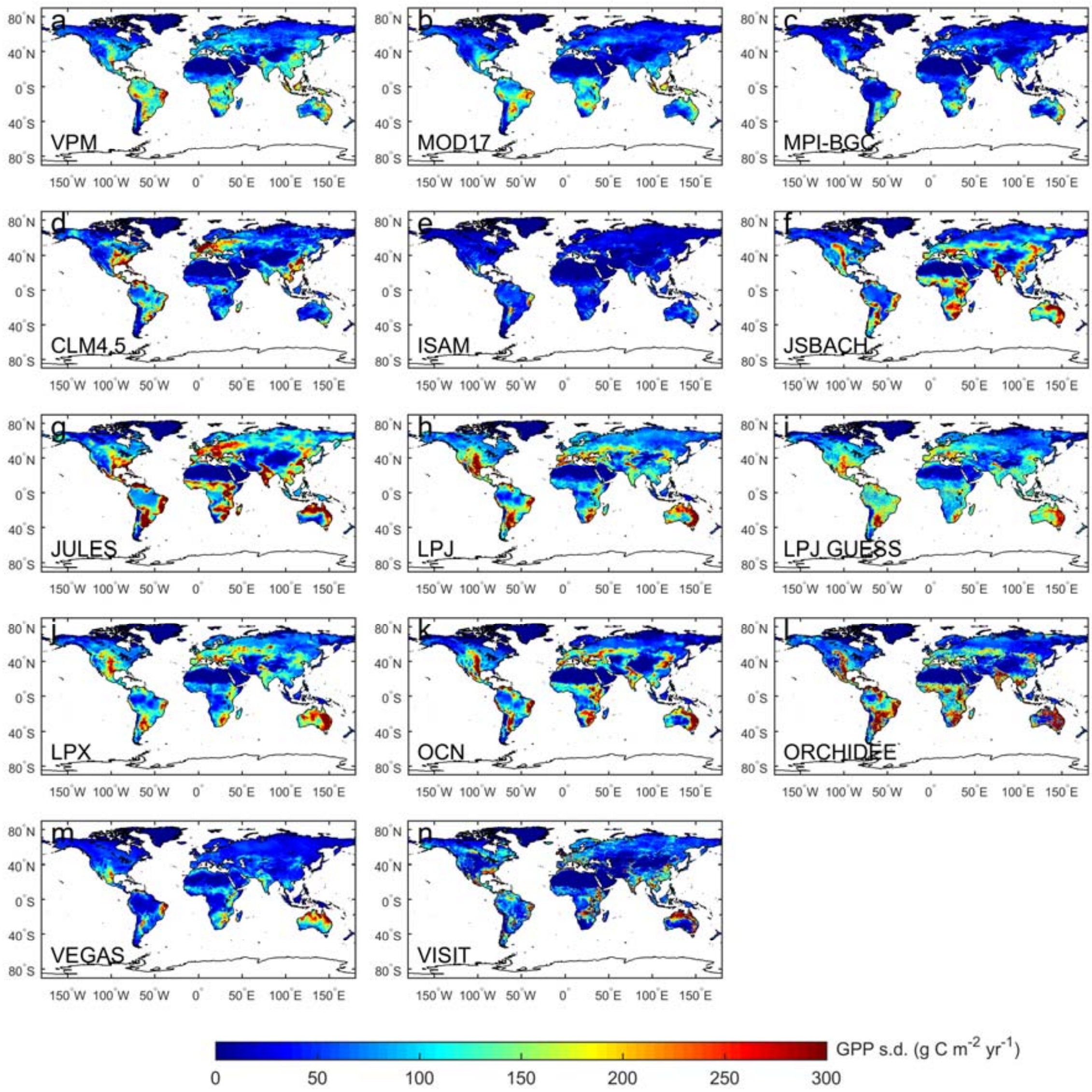

Fig. S3. Spatial patterns of inter-annual variability (standard deviation, s.d.) of GPP from the 3 data-driven models and the 11 DGVMs from the Trendy v4 project. Maps were created using Matlab R2016a (http://www.mathworks.com/products/matlab/).

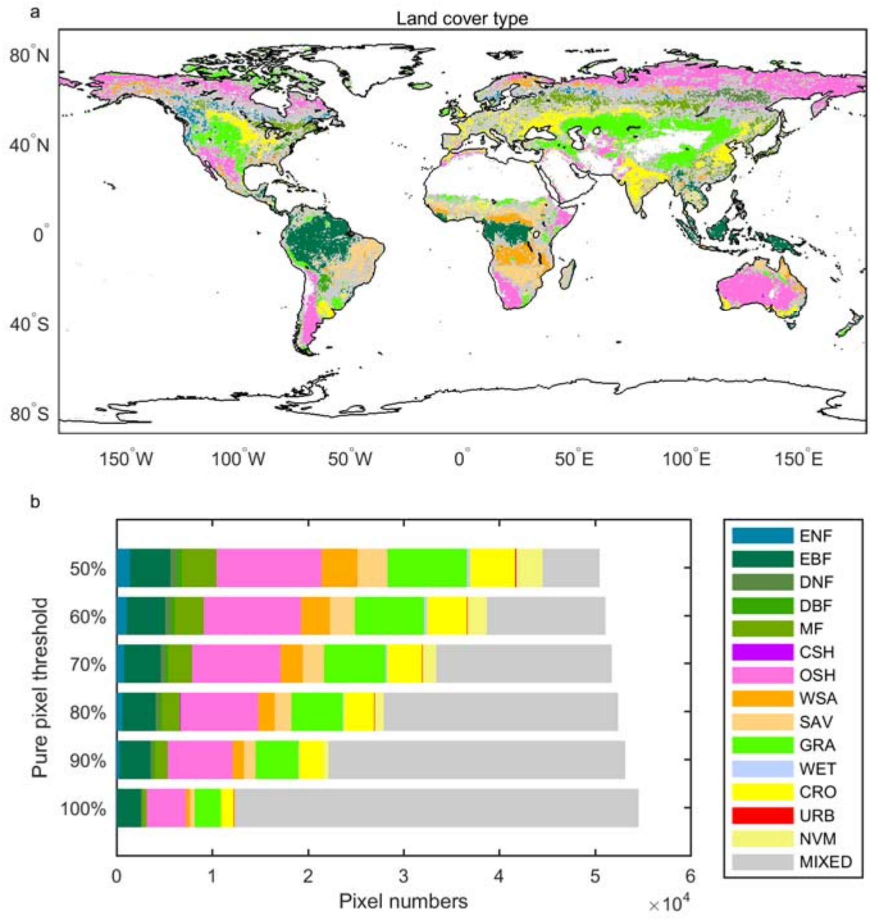

Fig. S4. Land cover map (80% threshold value) from MODIS MCD12Q1 for 2007 (a) and the 'pure' pixel numbers for each land cover type at different threshold values(b). For statistic purpose, only vegetated land (no snow, barren, water) were shown. Maps were created using Matlab R2016a (http://www.mathworks.com/products/matlab/).

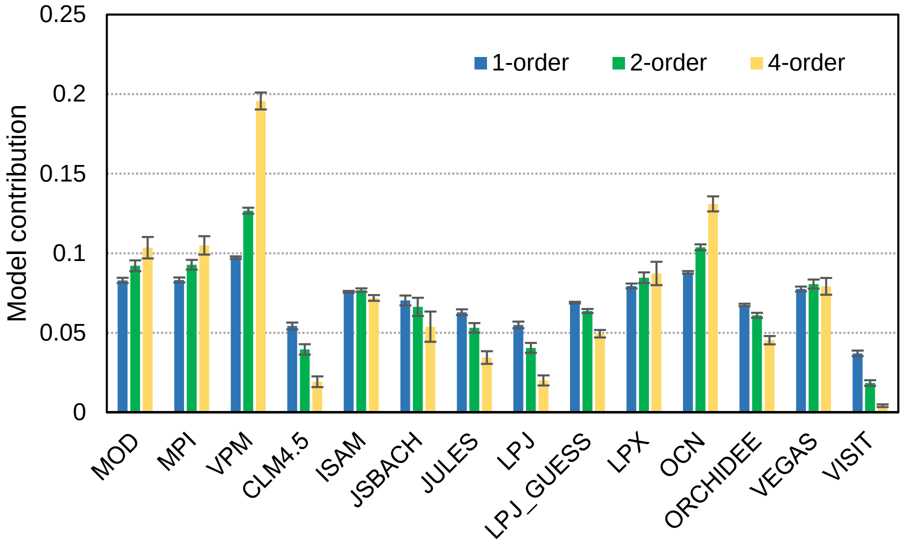

Fig. S5. Average model contribution of spatial GPP ensemble (see Methods) with different orders. The error bars represent the standardized deviation across 5 years (2007~2011).

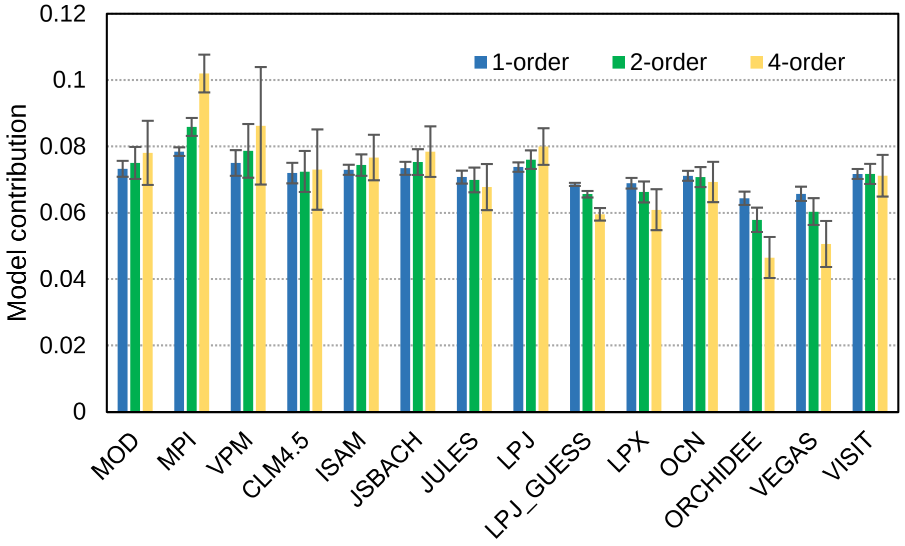

Fig. S6. Average model contribution of temporal GPP ensemble (see Methods) with different orders. The error bars represent the standardized deviation (s.d.) across 5 years (2007~2011).

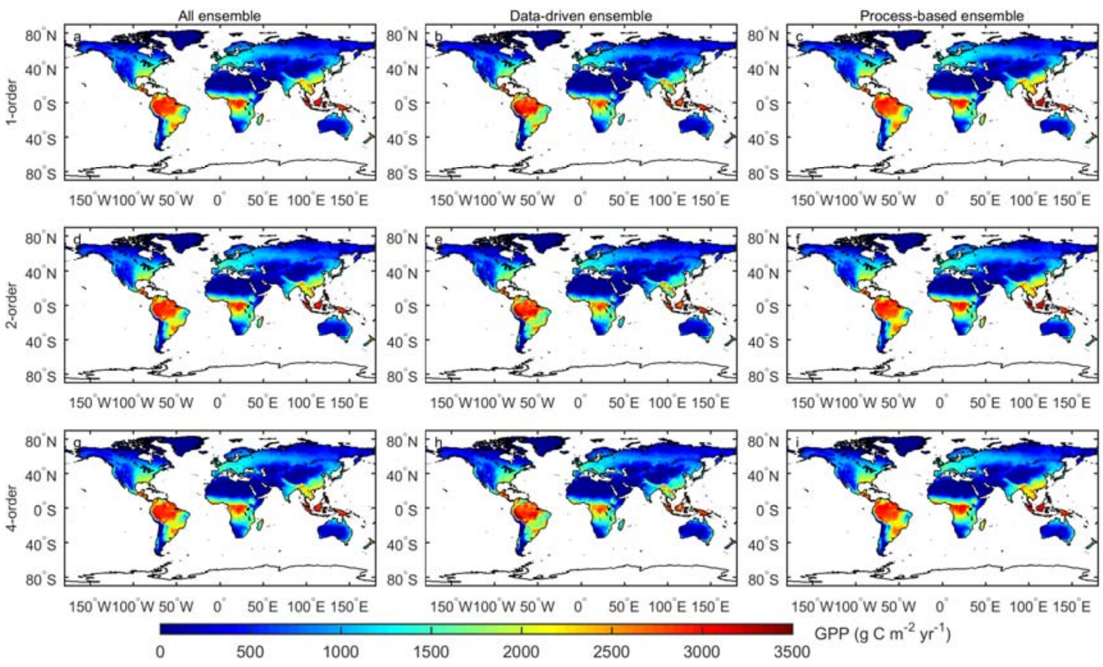

Fig. S7. Spatial pattern of annual GPP ensemble from all models (first column), data-driven models (second column), and DGVMs (third column). Different rows represent different orders of weight applied. Maps were created using Matlab R2016a (http://www.mathworks.com/products/matlab/).

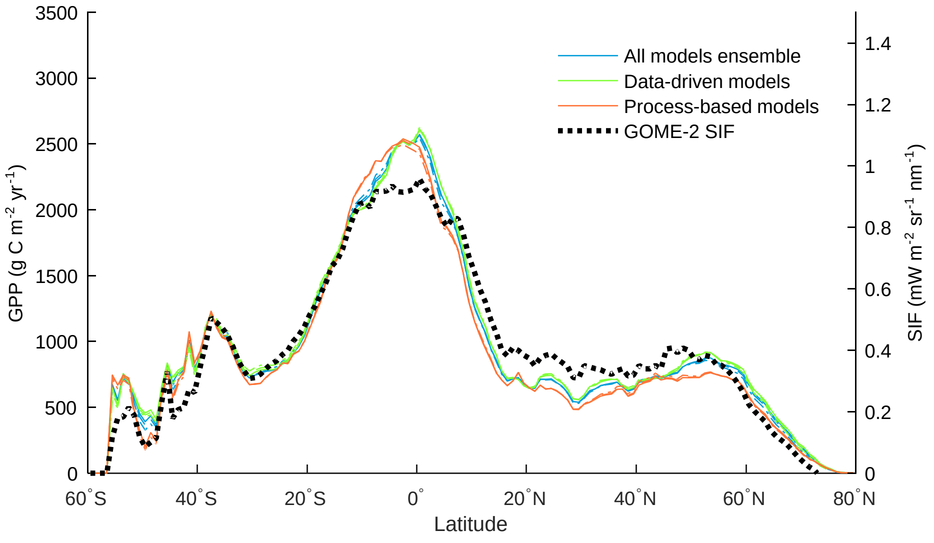

Fig. S8. Latitudinal pattern (1° bands) of annual GPP. The solid line, dashed line and dash-dotted line represent 1-order, 2-order and 4-order weighted ensemble.

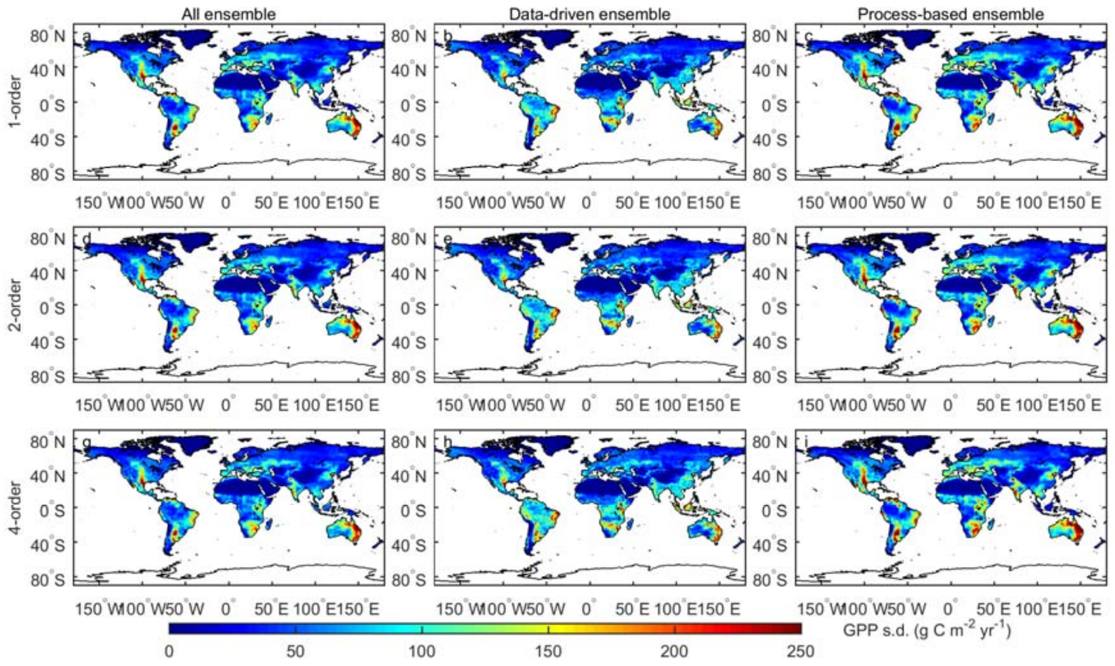

Fig. S9. Spatial pattern of GPP IAV from all models ensemble (first column), data-driven models (second column), DGVMs (third column). Different rows represent different orders of weight applied. Maps were created using Matlab R2016a (http://www.mathworks.com/products/matlab/).

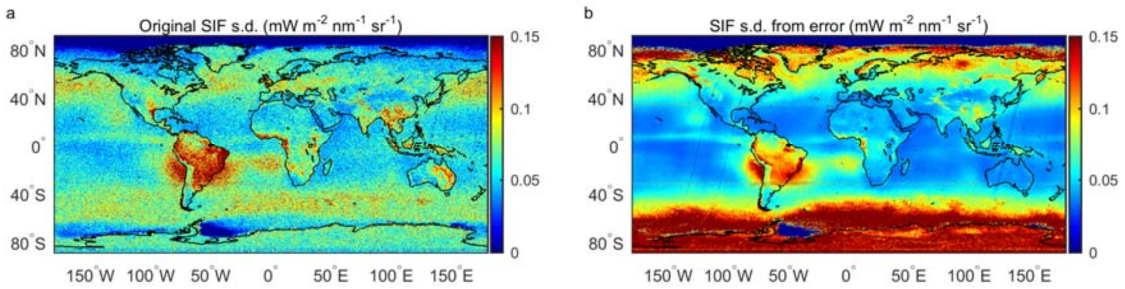

Fig. S10. The calculated SIF variability from 2007 to 2015 using annual mean SIF value (a) and the average SIF error from each year (b). Maps were created using Matlab R2016a (http://www.mathworks.com/products/matlab/).

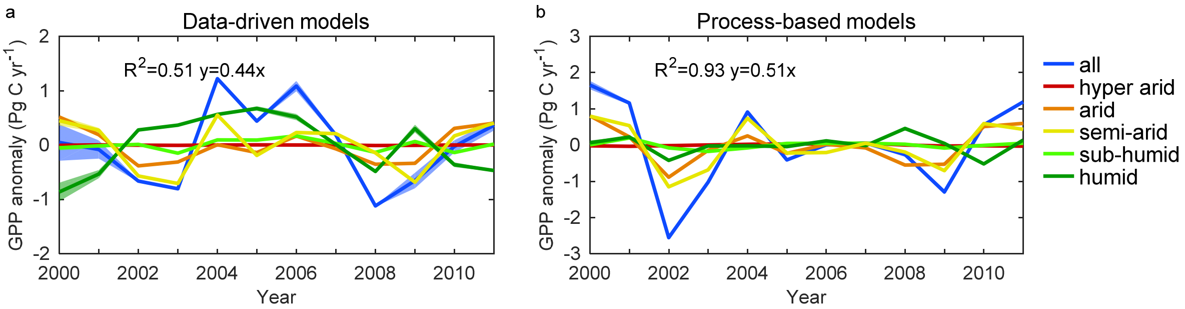

Fig. S11. Detrended GPP anomaly from the ensembles of data-driven models (a) and process-based models (b).

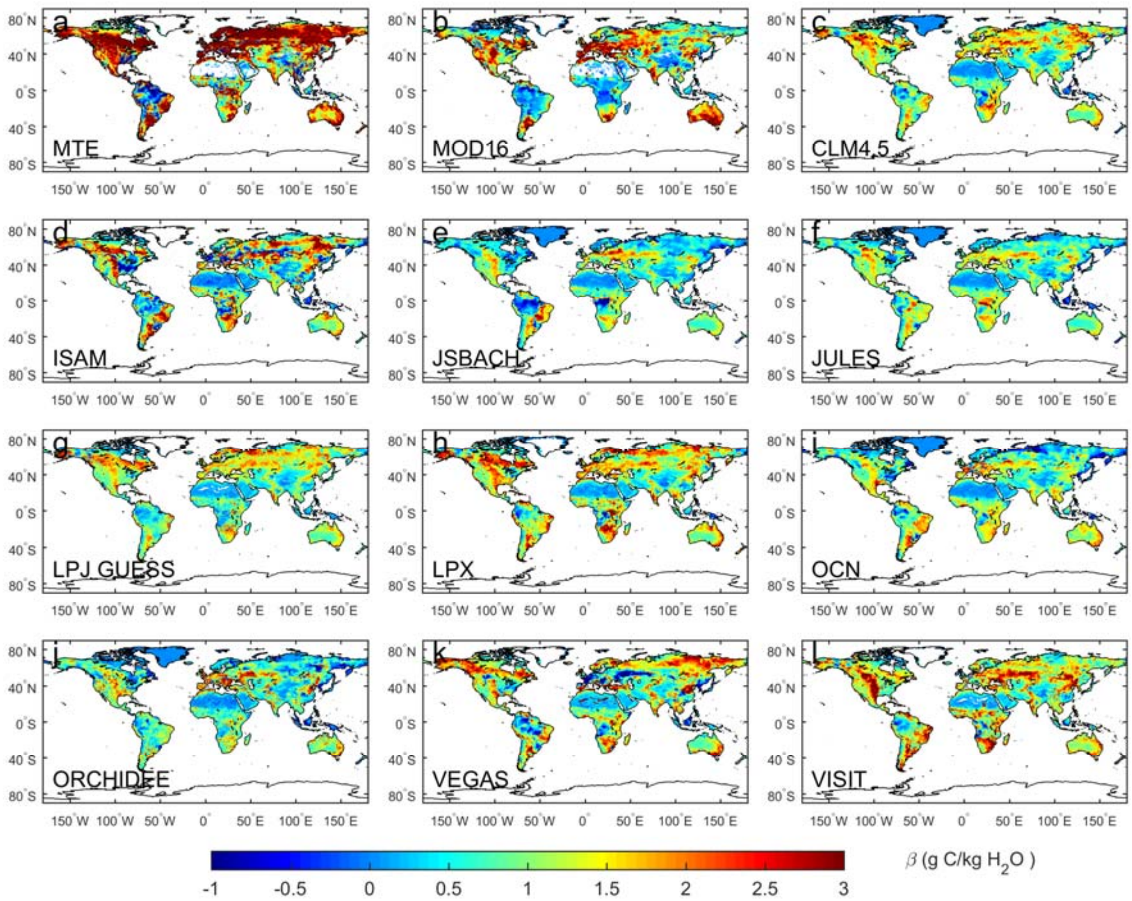

Fig. S12. Regression slope between GPP and ET. GPP is from 2nd order of the GPP ensemble with all models and ET is from 12 models. Maps were created using Matlab R2016a (http://www.mathworks.com/products/matlab/).

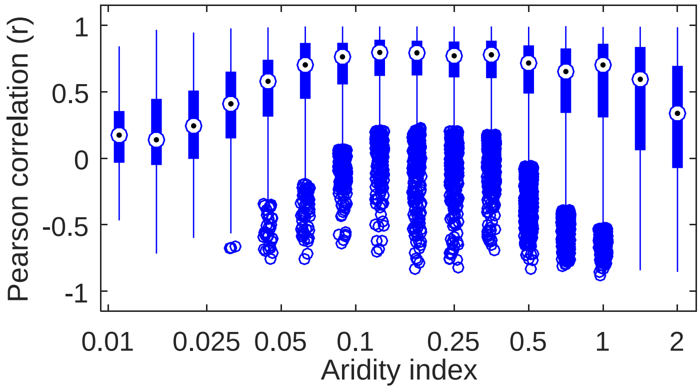

Fig. S13. Correlation distribution between 2nd order GPP ensemble and MTE ET across different aridity index. The water-carbon cycle is closely coupled in arid and semi-arid regions, but decreases after aridity index is greater than 0.5.

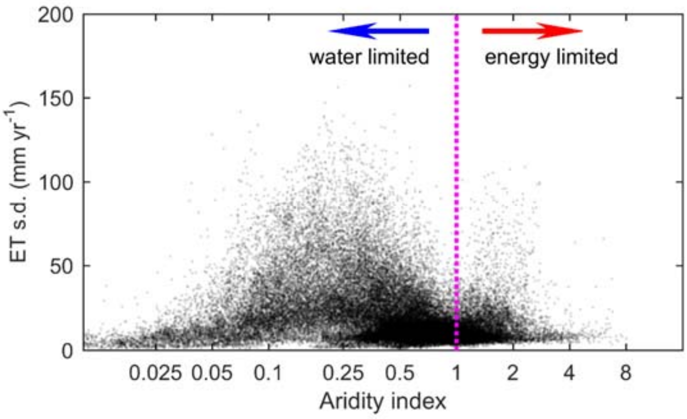

Fig. S14. Relationship between ET variability and aridity index. ET variation are calculated from MTE ET dataset and aggregated to annual sum. Aridity equals to 1 indicate precipitation equals to evapotranspirative demand. With the aridity index decreasing from 1 to 0, ET variability becomes dominated by the precipitation variability; with the aridity index increasing from 1 to ∞, ET variability becomes dominated by the PET variability.

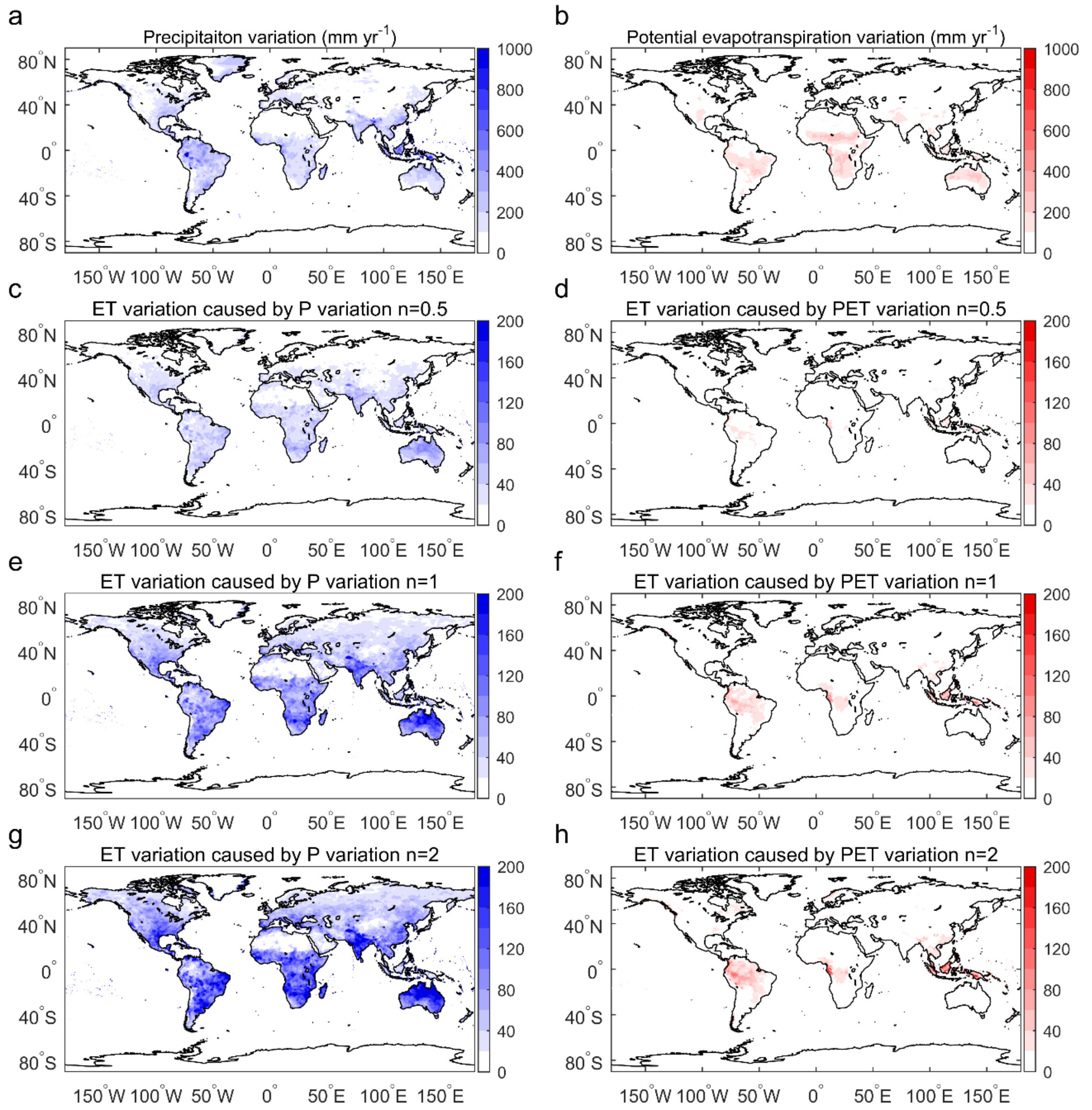

Fig. S15. The spatial pattern of precipitation variation from GPCC and PET variation from MODIS MOD16, both datasets are annual sums from 2000-2013 (a, b). ET variation caused by precipitation variation and by PET variation with different *n* values (c - h). Maps were created using Matlab R2016a (http://www.mathworks.com/products/matlab/).

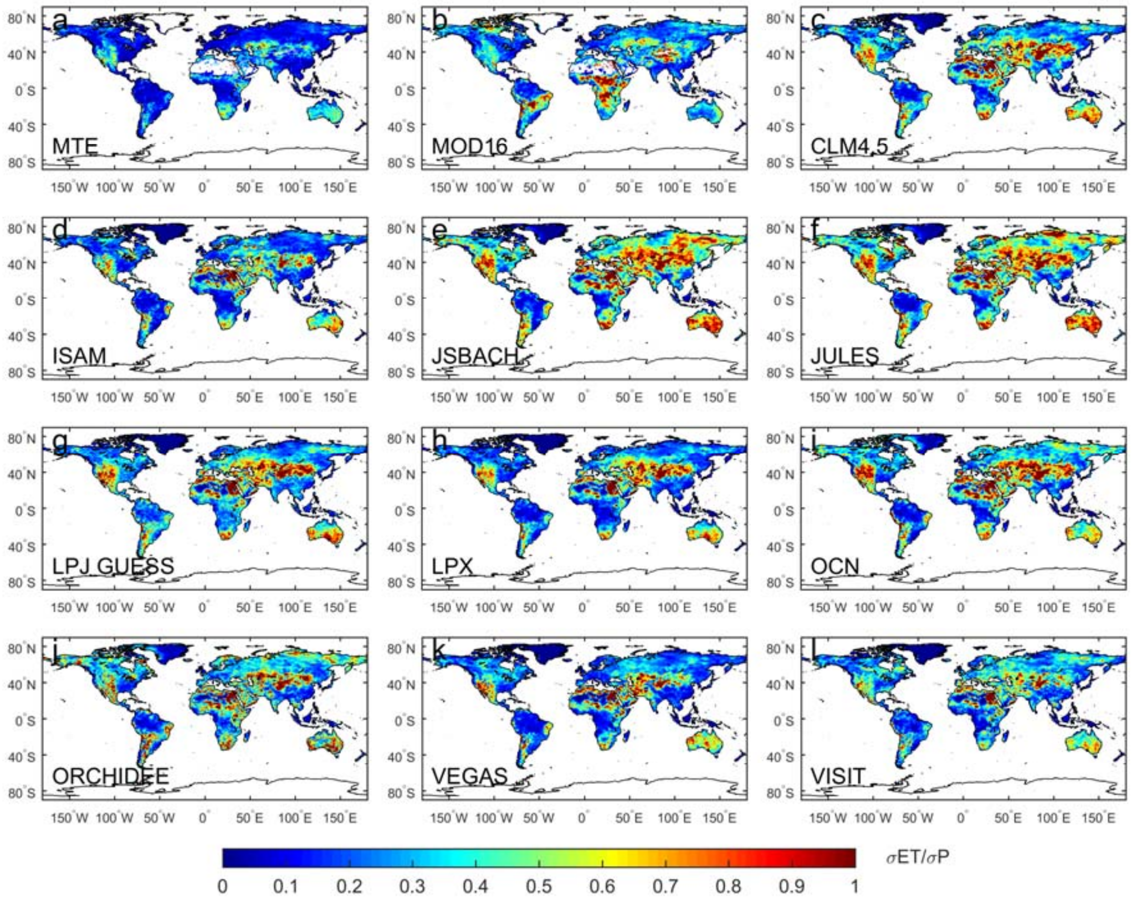

Fig. S16. σ_{ET}/σ_P estimated from 12 ET models and the GPCC precipitation for 2000-2011. Maps were created using Matlab R2016a (http://www.mathworks.com/products/matlab/).

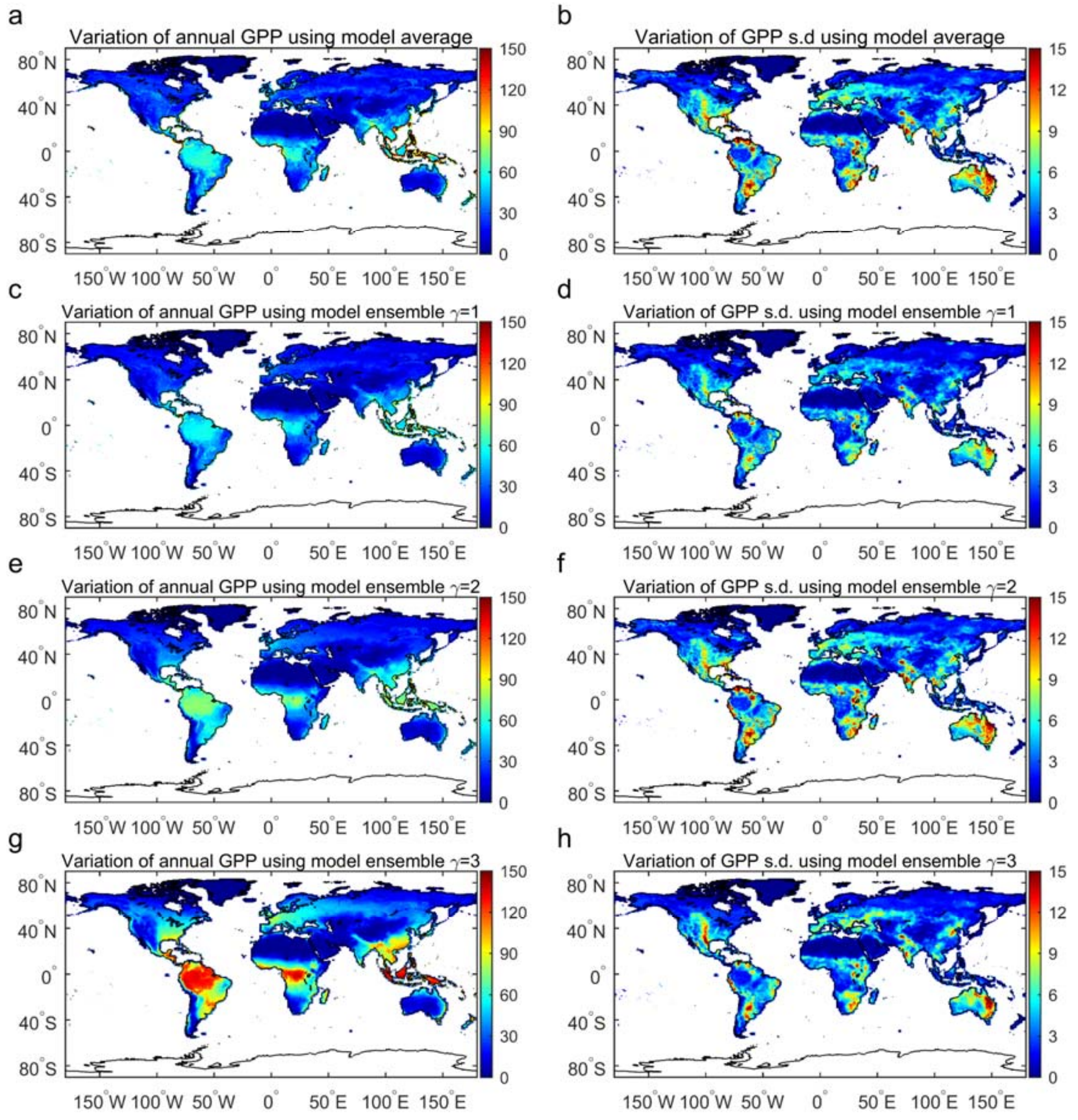

Fig. S17. The variation of (a, c, e, g) annual GPP and (b, d, f, h) GPP s.d. using unweighted average (a, b) and weighted ensemble (c, d, e, f, g, h) with different order factor (γ in Equation 3). All the units are g C m^{-2} yr^{-1}. Maps were created using Matlab R2016a (http://www.mathworks.com/products/matlab/).

Table S1. Classification scheme of aridity regions based on aridity index (AI). Aridity index is defined by FAO and the classification is inherited from UNEP Drylands are defined as AI < 0.65.

ARIDITY INDEX	ARIDITY CLASS
**<0.05**	Hyper arid
**0.05 – 0.2**	Arid
**0.2 – 0.5**	Semi-arid
**0.5 – 0.65**	Sub-humid
**>0.65**	Humid

Table S2. The biome area weighted correlation between GPP and SIF (score_i) using SIF as a temporal reference.

MODELS	2007	2008	2009	2010	2011
**VPM**	0.91	0.90	0.94	0.87	0.88
**MOD**	0.87	0.90	0.88	0.90	0.85
**MPI**	0.94	0.96	0.93	0.94	0.95
**CLM4.5**	0.87	0.91	0.77	0.89	0.89
**ISAM**	0.87	0.92	0.82	0.88	0.90
**JSBACH**	0.86	0.89	0.88	0.87	0.91
**JULES**	0.82	0.89	0.83	0.84	0.88
**LPJ**	0.87	0.92	0.87	0.87	0.91
**LPJG**	0.83	0.86	0.79	0.82	0.82
**LPX**	0.84	0.85	0.78	0.85	0.82
**OCN**	0.84	0.89	0.81	0.85	0.89
**ORCHIDEE**	0.78	0.81	0.71	0.81	0.77
**VEGAS**	0.81	0.84	0.73	0.80	0.77
**VISIT**	0.89	0.87	0.82	0.86	0.87

Table S3. The biome area weighted correlation between GPP and SIF (score_i) using SIF as a spatial reference.

MODELS	2007	2008	2009	2010	2011
**VPM**	0.71	0.74	0.74	0.76	0.74
**MOD**	0.59	0.63	0.63	0.66	0.63
**MPI**	0.60	0.63	0.62	0.66	0.64
**CLM4.5**	0.42	0.41	0.41	0.42	0.39
**ISAM**	0.55	0.57	0.57	0.59	0.58
**JSBACH**	0.51	0.51	0.51	0.59	0.53
**JULES**	0.47	0.45	0.47	0.51	0.48
**LPJ**	0.41	0.43	0.43	0.41	0.40
**LPJG**	0.51	0.52	0.52	0.54	0.51
**LPX**	0.59	0.62	0.59	0.62	0.59
**OCN**	0.65	0.67	0.66	0.68	0.67
**ORCHIDEE**	0.50	0.51	0.51	0.52	0.51
**VEGAS**	0.56	0.57	0.58	0.62	0.59
**VISIT**	0.28	0.28	0.30	0.28	0.27

Reference

1. Xiao X*, et al.* (2004) Satellite-based modeling of gross primary production in an evergreen needleleaf forest. *Remote Sensing of Environment* 89(4):519-534.
2. Xiao X*, et al.* (2004) Modeling gross primary production of temperate deciduous broadleaf forest using satellite images and climate data. *Remote Sensing of Environment* 91(2):256-270.
3. Wagle P*, et al.* (2014) Sensitivity of vegetation indices and gross primary production of tallgrass prairie to severe drought. *Remote Sensing of Environment* 152:1-14.
4. Jin C*, et al.* (2015) Effects of in-situ and reanalysis climate data on estimation of cropland gross primary production using the Vegetation Photosynthesis Model. *Agricultural and Forest Meteorology* 213:240-250.
5. Jin C*, et al.* (2013) Phenology and gross primary production of two dominant savanna woodland ecosystems in Southern Africa. *Remote Sensing of Environment* 135:189-201.
6. Kross A, Seaquist JW, & Roulet NT (2016) Light use efficiency of peatlands: Variability and suitability for modeling ecosystem production. *Remote Sensing of Environment* 183:239-249.
7. Sims DA*, et al.* (2006) On the use of MODIS EVI to assess gross primary productivity of North American ecosystems. *J Geophys Res-Biogeo* 111(G4).
8. Sims D*, et al.* (2008) A new model of gross primary productivity for North American ecosystems based solely on the enhanced vegetation index and land surface temperature from MODIS. *Remote Sensing of Environment* 112(4):1633-1646.
9. Zhang Y*, et al.* (2016) Canopy and physiological controls of GPP during drought and heat wave. *Geophysical Research Letters* 43(7):3325-3333.
10. Zhang Y*, et al.* (2016) Consistency between sun-induced chlorophyll fluorescence and gross primary

production of vegetation in North America. *Remote Sensing of Environment* 183:154-169.

11. Jung M, Reichstein M, & Bondeau A (2009) Towards global empirical upscaling of FLUXNET eddy covariance observations: validation of a model tree ensemble approach using a biosphere model. *Biogeosciences* 6(10):2001-2013.
12. Jung M*, et al.* (2011) Global patterns of land-atmosphere fluxes of carbon dioxide, latent heat, and sensible heat derived from eddy covariance, satellite, and meteorological observations. *J Geophys Res-Biogeo* 116.
13. Sitch S*, et al.* (2015) Recent trends and drivers of regional sources and sinks of carbon dioxide. *Biogeosciences* 12(3):653-679.
14. Oleson K*, et al.* (2013) Technical description of version 4.5 of the Community Land Model (CLM). NCAR Tech. (Note NCAR/TN-503+ STR. National Center for Atmospheric Research, Boulder, CO, 422 pp. doi: 10.5065/D6RR1W7M).
15. Kato E, Kinoshita T, Ito A, Kawamiya M, & Yamagata Y (2013) Evaluation of spatially explicit emission scenario of land-use change and biomass burning using a process-based biogeochemical model. *Journal of Land Use Science* 8(1):104-122.
16. Raddatz T*, et al.* (2007) Will the tropical land biosphere dominate the climate–carbon cycle feedback during the twenty-first century? *Climate Dynamics* 29(6):565-574.
17. Clark DB*, et al.* (2011) The Joint UK Land Environment Simulator (JULES), model description – Part 2: Carbon fluxes and vegetation dynamics. *Geoscientific Model Development* 4(3):701-722.
18. Sitch S*, et al.* (2003) Evaluation of ecosystem dynamics, plant geography and terrestrial carbon cycling in the LPJ dynamic global vegetation model. *Global change biology* 9(2):161-185.
19. Smith B, Prentice IC, & Sykes MT (2001) Representation of vegetation dynamics in the modelling of terrestrial ecosystems: comparing two contrasting approaches within European climate space. *Global Ecology and Biogeography* 10(6):621-637.
20. Prentice I*, et al.* (2011) Modeling fire and the terrestrial carbon balance. *Global Biogeochemical Cycles* 25(3).
21. Zaehle S & Friend AD (2010) Carbon and nitrogen cycle dynamics in the O-CN land surface model: 1. Model description, site-scale evaluation, and sensitivity to parameter estimates. *Global Biogeochemical Cycles* 24.
22. Krinner G*, et al.* (2005) A dynamic global vegetation model for studies of the coupled atmosphere-biosphere system. *Global Biogeochemical Cycles* 19(1).
23. Zeng N, Mariotti A, & Wetzel P (2005) Terrestrial mechanisms of interannual CO(2) variability. *Global Biogeochemical Cycles* 19(1).
24. Ito A (2008) The regional carbon budget of East Asia simulated with a terrestrial ecosystem model and validated using AsiaFlux data. *Agricultural and forest meteorology* 148(5):738-747.
25. Joiner J, Yoshida Y, Guanter L, & Middleton E (2016) New methods for retrieval of chlorophyll red fluorescence from hyper-spectral satellite instruments: simulations and application to GOME-2 and SCIAMACHY. *Atmospheric Measurement Techniques*.
26. Joiner J*, et al.* (2013) Global monitoring of terrestrial chlorophyll fluorescence from moderate-spectral-resolution near-infrared satellite measurements: methodology, simulations, and application to GOME-2. *Atmospheric Measurement Techniques* 6(10):2803-2823.
27. Mu QZ, Zhao MS, & Running SW (2011) Improvements to a MODIS global terrestrial evapotranspiration algorithm. *Remote Sensing of Environment* 115(8):1781-1800.
28. Mu Q, Heinsch FA, Zhao M, & Running SW (2007) Development of a global evapotranspiration algorithm

based on MODIS and global meteorology data. *Remote Sensing of Environment* 111(4):519-536.

29. Schneider U*, et al.* (2011) GPCC full data reanalysis version 6.0 at 0.5: monthly land-surface precipitation from rain-gauges built on GTS-based and historic data. doi: 10.5676/DWD_GPCC. *FD_M_V6_050*.

30. Harris I, Jones P, Osborn T, & Lister D (2014) Updated high - resolution grids of monthly climatic observations – the CRU TS3. 10 Dataset. *International Journal of Climatology* 34(3):623-642.

31. Middleton N & Thomas D (1997) *World atlas of desertification* (Arnold, Hodder Headline, PLC).

32. Frankenberg C*, et al.* (2011) New global observations of the terrestrial carbon cycle from GOSAT: Patterns of plant fluorescence with gross primary productivity. *Geophysical Research Letters* 38.

33. Guanter L*, et al.* (2012) Retrieval and global assessment of terrestrial chlorophyll fluorescence from GOSAT space measurements. *Remote Sensing of Environment* 121:236-251.

34. Damm A*, et al.* (2015) Far-red sun-induced chlorophyll fluorescence shows ecosystem-specific relationships to gross primary production: An assessment based on observational and modeling approaches. *Remote Sensing of Environment* 166:91-105.

35. van der Tol C, Verhoef W, Timmermans J, Verhoef A, & Su Z (2009) An integrated model of soil-canopy spectral radiances, photosynthesis, fluorescence, temperature and energy balance. *Biogeosciences* 6(12):3109-3129.

36. Lee JE*, et al.* (2015) Simulations of chlorophyll fluorescence incorporated into the Community Land Model version 4. *Global change biology*.

37. van der Tol C, Berry JA, Campbell PKE, & Rascher U (2014) Models of fluorescence and photosynthesis for interpreting measurements of solar-induced chlorophyll fluorescence. *J Geophys Res-Biogeo* 119(12):2312-2327.
